# Sialylation of Prion Protein Controls the Rate of Prion Amplification, the Cross-Species Barrier, the Ratio of PrP^Sc^ Glycoform and Prion Infectivity

**DOI:** 10.1371/journal.ppat.1004366

**Published:** 2014-09-11

**Authors:** Elizaveta Katorcha, Natallia Makarava, Regina Savtchenko, Alessandra d′Azzo, Ilia V. Baskakov

**Affiliations:** 1 Center for Biomedical Engineering and Technology, University of Maryland School of Medicine, Baltimore, Maryland, United States of America; 2 Department of Anatomy and Neurobiology, University of Maryland School of Medicine, Baltimore, Maryland, United States of America; 3 Department of Genetics, St. Jude Children's Research Hospital, Memphis, Tennessee, United States of America; University of Florida, United States of America

## Abstract

The central event underlying prion diseases involves conformational change of the cellular form of the prion protein (PrP^C^) into the disease-associated, transmissible form (PrP^Sc^). PrP^C^ is a sialoglycoprotein that contains two conserved N-glycosylation sites. Among the key parameters that control prion replication identified over the years are amino acid sequence of host PrP^C^ and the strain-specific structure of PrP^Sc^. The current work highlights the previously unappreciated role of sialylation of PrP^C^ glycans in prion pathogenesis, including its role in controlling prion replication rate, infectivity, cross-species barrier and PrP^Sc^ glycoform ratio. The current study demonstrates that undersialylated PrP^C^ is selected during prion amplification in Protein Misfolding Cyclic Amplification (PMCAb) at the expense of oversialylated PrP^C^. As a result, PMCAb-derived PrP^Sc^ was less sialylated than brain-derived PrP^Sc^. A decrease in PrP^Sc^ sialylation correlated with a drop in infectivity of PMCAb-derived material. Nevertheless, enzymatic de-sialylation of PrP^C^ using sialidase was found to increase the rate of PrP^Sc^ amplification in PMCAb from 10- to 10,000-fold in a strain-dependent manner. Moreover, de-sialylation of PrP^C^ reduced or eliminated a species barrier of for prion amplification in PMCAb. These results suggest that the negative charge of sialic acid controls the energy barrier of homologous and heterologous prion replication. Surprisingly, the sialylation status of PrP^C^ was also found to control PrP^Sc^ glycoform ratio. A decrease in PrP^C^ sialylation levels resulted in a higher percentage of the diglycosylated glycoform in PrP^Sc^. 2D analysis of charge distribution revealed that the sialylation status of brain-derived PrP^C^ differed from that of spleen-derived PrP^C^. Knocking out lysosomal sialidase Neu1 did not change the sialylation status of brain-derived PrP^C^, suggesting that Neu1 is not responsible for desialylation of PrP^C^. The current work highlights previously unappreciated role of PrP^C^ sialylation in prion diseases and opens multiple new research directions, including development of new therapeutic approaches.

## Author Summary

The central event underlying prion diseases involves conformational change of the cellular form of the prion protein (PrP^C^) into disease-associated, transmissible form (PrP^Sc^). The amino acid sequence of PrP^C^ and strain-specific structure of PrP^Sc^ are among the key parameters that control prion replication and transmission. The current study showed that PrP^C^ posttranslational modification, specifically sialylation of N-linked glycans, plays a key role in regulating prion replication rate, infectivity, cross-species barrier and PrP^Sc^ glycoform ratio. A decrease in PrP^C^ sialylation level increased the rate of prion replication in a strain-specific manner and reduced or eliminated a species barrier when prion replication was seeded by heterologous seeds. At the same time, a decrease in sialylation correlated with a drop in infectivity of PrP^Sc^ material produced *in vitro*. The current study also demonstrated that the PrP^Sc^ glycoform ratio, which is an important feature used for strain typing, is not only controlled by prion strain or host but also the sialylation status of PrP^C^. This study opens multiple new directions in prion research, including development of new therapeutic approaches.

## Introduction

Prion disease is a family of lethal, neurodegenerative maladies that can be sporadic, inheritable or transmissible in origin [1]. The key molecular event underlying prion diseases involves conformational change of the normal, cellular form of the prion protein denoted PrP^C^ into the disease-associated, self-propagating, transmissible form denoted PrP^Sc^ [2]. Upon expression in the endoplasmic reticulum, PrP^C^ undergoes posttranslational modifications, including attachment of up to two N-linked carbohydrates to residues Asn-181 and Asn-197 and of glycosylinositol phospholipid anchor (GPI) to the C-terminal residue Ser-231 (residue numbers are given for hamster PrP^C^) [3]–[5]. These posttranslational modifications are intact upon conversion of PrP^C^ into PrP^Sc^ [4], [6], [7].

Since the discovery that the PrP^Sc^ and PrP^C^ glycans are sialylated more than 25 years ago [6], [8], the potential role of sialylation in PrP^C^ function, prion replication or its pathogenesis remains uncertain. The two N-linked carbohydrates can carry from zero to four terminal sialic acid residues each [8], [9]. While the PrP polypeptide has a strong positive charge, the isoelectric points (pI) of PrP^C^ can vary significantly in part due to variation in sialylation of the glycans [10]–[15]. In glycans sialic acid residues are linked to the galactose residues at the C-6 or C-3 positions [8]. The detailed site-specific characterization of mouse PrP^C^ revealed that the majority of glycans at Ans-180 have bi- and triantennary structures and are sialylated to a lesser degree than the glycans at Ans-196, a majority of which are tri- and tetraantennary structures [16]. While the relative proportion of bi-, tri-, and tetra-antennary glycans appears to differ slightly in PrP^C^ and PrP^Sc^, the relative proportions of sialylated glycans was found not to be statistically different between PrP^C^ and PrP^Sc^ [9]. Due to diverse structure and composition of oligosaccharides, PrP^C^ primary structure consists of more than 400 different glycoforms. In addition to sialylation of both glycans, a single sialic acid was also found on a GPI anchor of PrP^C^ and PrP^Sc^ [3].

The ratio of di-, mono-, and unglycosylated PrP^C^ glycoforms was found to change in favor of di-glycosylated forms in the course of neuronal differentiation, as well as upon an increase in the density of cells cultured *in vitro* [17], [18]. While diglycosylated PrP^C^ is the dominant glycoform in adult brain, the ratio of di-, mono-, and unglycosylated PrP^C^ glycoforms was found to vary in different brain regions [19]. 2D-gel electrophoresis analysis revealed variations in isoelectric points (pI) of PrP^C^ isoforms expressed in different brain regions [10], a variation that could presumably be attributed, at least in part, to the region-specific differences in sialylation status of glycans. Moreover, as probed by binding of 19 lectins specific to different sugars including sialic acid residues, the composition of PrP^C^ glycans was found to change with normal aging [20].

In the last decade, numerous studies illustrated the essential role of protein sialylation in immunity including its role in cell signaling, cell activation, differentiation, and pathogen recognition (reviewed in [21]-[23]). While sialylation of cell surface proteins is also involved in a number of functions of central nervous system, including cell differentiation, adhesion and neuronal plasticity, a big gap in understanding the role of sialylation in the normal and pathological function of PrP^C^ exists. Sialylation of PrP^C^ glycans was shown to prevent binding of PrP^C^ to selectins, a family of cell surface proteins that interact with carbohydrates in a Ca^2+^-dependent manner and participate in cell adhesion and migration [24]. A recent study examined the role of Siglec-1, a sialic acid-binding immunoglobulin-type lectin, expression of which is restricted to mononuclear phagocytes, in prion diseases [25]. While mononuclear phagocytes are known to be important for prion uptake and trafficking to/within lymphoid tissue and possibly prion clearance, no effect of Siglec-1 knockout on peripheral prion disease pathogenesis was observed [25]. Another study examined possible involvement of GPI sialylation in neurodegeneration and found that a dense clustering of sialic acid-containing GPI anchors in the plasma membrane resulted in alteration of membrane composition and synapse damage [26], [27]. The presence of sialic acid in the GPI was a requirement for the toxic effect expressed by clustering of PrP^C^ molecules on cell surface [26], [27].

In the current work, we examined the role of sialylation of PrP^C^ on prion replication, a topic that has not been addressed in previous studies. We showed that charge heterogeneity in brain-derived PrP^C^ and PrP^Sc^ was due to sialylation and that undersialylated PrP^C^ molecules (sialylated less than the statistical average for PrP^C^) were a preferable substrate for prion amplification in PMCAb. As a result, PrP^Sc^ produced in PMCAb was less sialylated than brain-derived PrP^Sc^ and also showed longer incubation time to disease. Consistent with the idea that sialylation of PrP^Sc^ is important for prion infectivity, PMCAb material produced using desialylated PrP^C^ was not infectious. Nevertheless, in support of the hypothesis that PrP^C^ sialylation controls prion replication rate, de-sialylation of PrP^C^ was found to speed up considerably PrP^Sc^ amplification in PMCAb, with the magnitude of this effect found to be strain-dependent. Moreover, de-sialylation of PrP^C^ reduced or eliminated a species barrier of prion amplification in PMCAb. Furthermore, 2D analysis suggested that sialylation status of brain-derived PrP^C^ was different from that of spleen-derived PrP^C^. Surprisingly, the PrP^Sc^ glycoform ratio was found to be controlled by the sialylation status of PrP^C^, with a decrease in PrP^C^ sialylation levels resulting in a higher percentage of the diglycosylated glycoform in PrP^Sc^ presumably due to a decrease in density of negatively charged groups on PrP^Sc^ surface. The current study exposes the previously underappreciated role of PrP^C^ sialylation in a number of key aspects of prion diseases, including its role in controlling prion replication rate, its infectivity, species barrier and PrP^Sc^ glycoform ratio.

## Results

### Charge heterogeneity in brain-derived PrP^C^ and PrP^Sc^ is due to sialylation

**Figure 1:**
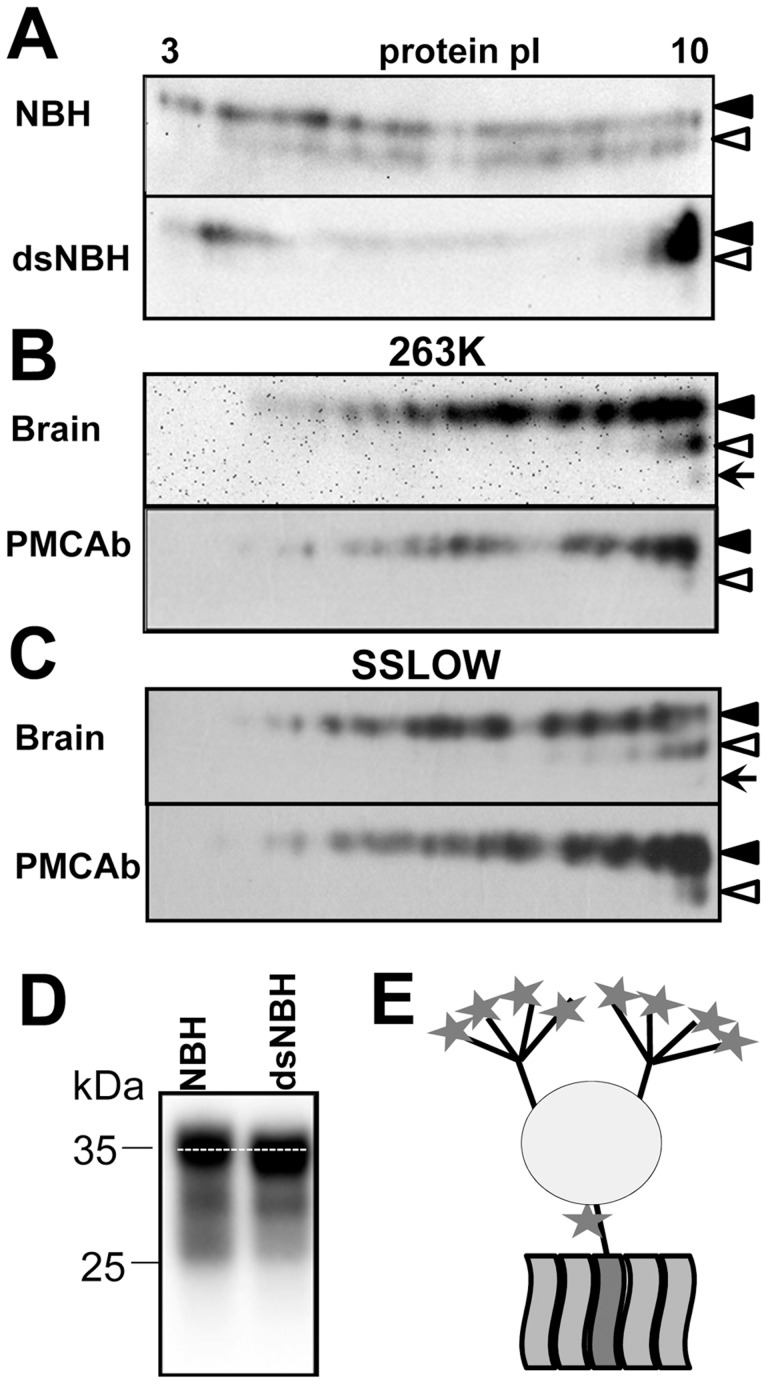
2D analysis of PrP^C^ and brain- and PMCAb-derived PrP^Sc^. **A.** 2D analysis of Syrian hamster NBH (top panel) and NBH treated with sialidase from *A. ureafaciens* (bottom gel). **B, C.** 2D analysis of brain- and PMCAb-derived 263K material (**B**), and brain- and PMCAb-derived SSLOW material (**C**). To produce PMCAb-derived material, brain-derived 263K or SSLOW was subjected to 24 serial rounds with 10-fold dilution between rounds. Black and white triangles mark diglycosylated and monoglycosylated glycoforms, respectively, whereas arrows mark the unglycosylated form. D. Migration of PrP^C^ in 1D SDS-PAGE gel before and after treatment with sialidase. All blots were stained with 3F4 antibody. **E.** Schematic diagram of PrP^C^ that illustrates location of sialic acid residues (stars) on N-linked glycans and GPI anchor. Each of the two glycans can carry up to four terminal sialic acid residues.

In the absence of posttranslational modifications the prion protein has a strong positive charge at physiological pH. Theoretical calculation of the isoelectric point for full-length Syrian hamster PrP predicts a value 9.58. However, due to posttranslational modifications and, primarily, sialylation of N-linked glycans and the GPI anchor, the actual isoelectric points of PrP^C^ molecules could be substantially lower than 9.58 [14]. In fact, because each of the two N-linked glycans contains up to four terminal sialic acids (Figure 1E), brain-derived PrP^C^ molecules are heterogeneous with respect to their charge as confirmed by 2D gel-electrophoresis (Figure 1A).

To test whether sialic acid residues indeed account for broad charge heterogeneity, Syrian hamster normal brain homogenate (NBH) was treated with *A. ureafaciens* sialidase (sialidase-treated NBH will be referred to as dsNBH), an enzyme that cleaves off terminal α2,3- and α2,6-linked sialic acid residues. While in non-treated NBH PrP^C^ molecules are spread between pI 3 and 10 (Figure 1A, top), enzymatic desialylation resulted in a substantial shift of PrP^C^ towards pI 10. A relatively intense spot at acidic pH in dsNBH appears to be due to aggregation of PrP^C^ at low pH. Nevertheless, this experiment illustrates that PrP^C^ charge heterogeneity is attributable at least in part to its variable sialylation status. Consistent with a previous study [20], the diglycosylated form of PrP^C^ in dsNBH migrated slightly faster on a 1D gel than that in non-treated NBH (Figure 1D).

Brain-derived, proteinase K (PK)-treated scrapie material from animals infected with the strains of natural or synthetic origin 263K or SSLOW [28], respectively, also showed broad charge heterogeneity on 2D gels (Figure 1B,C). When compared to the 2D profile of PrP^C^, the charge distributions of PK-treated 263K and SSLOW were found to shift toward pI 10, despite an expected shift toward acidic pH due to proteolytic cleavage of the positively charged N-terminal region. The reason behind such a shift is difficult to explain. There is a possibility that PrP^Sc^ is less sialylated than PrP^C^, although no notable differences in sialylation status of PrP^C^ and PrP^Sc^ were found in previous study [9]. Alternatively, a fraction of PrP^C^ molecules could be subjected to posttranslational modifications including deamidation of Asn and Gln to Asp and Glu [29], [30], respectively, phosphorylation of serine 43 [15], or modification of amino groups of Lys and Arg by reducing sugars resulting in advanced glycation end-products [5], [31]. Such modification would increase PrP^C^ charge heterogeneity and account for spreading PrP^C^ to acidic pH on 2D. Attempts to remove sialic acid in PrP^Sc^ by treatment with sialidase were not successful, presumably due to high aggregation status of PrP^Sc^ (data not shown).

### Undersialylated PrP^C^ molecules are preferable substrate for prion amplification in PMCAb

Because previous studies showed that properties of PrP^Sc^ change during PMCA [32], [33], we decided to compare charge distribution of PMCAb-derived and brain-derived PrP^Sc^. To rule out any interference of the initial brain-derived PrP^Sc^ seeds, twenty four serial PMCAb rounds were performed with a dilution 1∶10 between rounds to produce PMCAb-derived material. Surprisingly, both 263K and SSLOW PMCAb-derived materials showed a considerable shift towards basic pI when compared to that of brain-derived PrP^Sc^ (Figure 1B,C). Moreover, consistent with the previous study [32], the percentage of monoglycosylated glycoforms decreased in PMCAb-derived material comparing to those of brain-derived PrP^Sc^. These results suggest that (i) undersialylated PrP^C^ molecules are selected during *in vitro* amplification at the expense of overersialylated PrP^C^ (sialylated more than the statistical average for PrP^C^) and (ii) a decrease in PrP^Sc^ sialylation level reduces the negative charge on PrP^Sc^ surfaces that might lead to an increase in percentage of diglycosyated molecules incorporated into PMCAb-derived material.

### Desialylation of PrP^C^ increases the rate of PrP^Sc^ amplification in a strain-dependent manner

**Figure 2:**
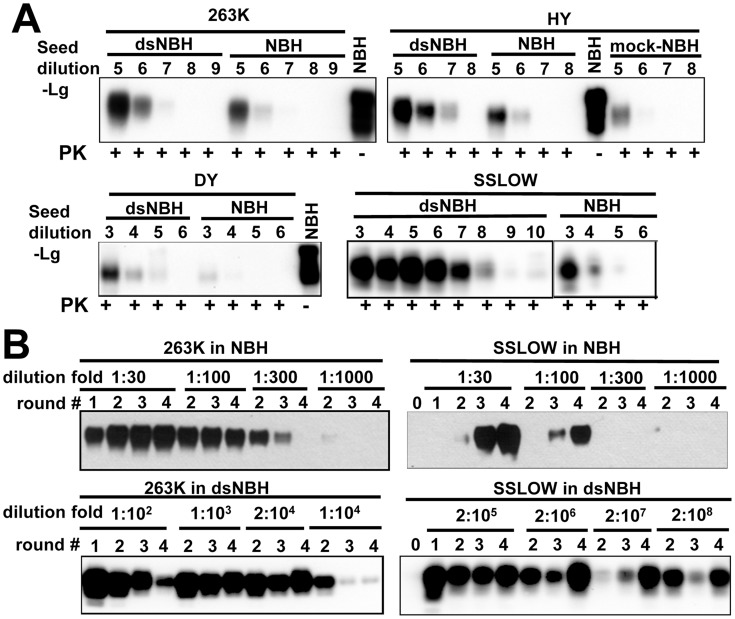
De-sialylation of PrP^C^ increases the rate of PrP^Sc^ amplification in PMCAb. **A.** 263K, HY, Drowsy or SSLOW scrapie brain materials were diluted 10^3^–10^10^-fold into 10% NBH, dsNBH or mock treated NBH (NBH incubated with a buffer for enzymatic de-sialylation in the absence of sialidase) as indicated and subjected to a single PMCAb round. Undigested 10% NBH was used as reference. **B.** Analysis of PrP^Sc^ amplification fold. Scrapie brain materials were diluted 10^4^-fold for 263K or 10^3^-fold for SSLOW into 10% NBH or dsNBH as indicated and subjected to four serial PMCAb rounds. The material amplified in each round was diluted to a specified dilution fold into 10% NBH or dsNBH for the next PMCAb round as indicated. Unamplified seeds are shown as round 0. Prior to electrophoresis, samples were treated with PK. All blots were stained with 3F4 antibody.

To provide independent support that undersialylated PrP^C^ is a preferable substrate for PrP^Sc^ amplification *in vitro*, we tested whether desialylation of PrP^C^ increases the rate of amplification using two alternative PMCAb formats. dsNBH prepared by treatment of NBH with *A.ureafaciens* sialidase (Figure 1A) was used as a substrate in PMCAb along with 10% non-treated NBH. In the first format, increasing dilutions of brain-derived 263K, Hyper, Drowsy and SSLOW materials were subjected to a single round of PMCAb conducted in dsNBH or NBH. The range of seed dilutions was chosen individually for each strain according to the previously published results [34]. For the hamster-adapted strains of natural origin (263K, Hyper (HY), Drowsy), the reactions conducted in dsNBH detected approximately 10-fold higher seed dilutions than the reactions conducted in NBH (Figure 2A). Surprisingly, for the synthetic strain SSLOW, the reaction conducted in dsNBH detected 10^4^-fold higher seed dilutions than the reactions conducted in NBH (Figure 2A). In fact, in dsNBH 10^8^-fold diluted SSLOW brain material was persistently detected in a single PMCAb round. For two other strains of synthetic origin LOTSS and S05 [35], [36], the amplification rate also increase by at least four orders of magnitude in dsNBH compared to that in NBH (data not shown).

In a second format, a set of serial PMCAb reactions were conducted for 263K or SSLOW in NBH or dsNBH with the dilution folds between serial rounds ranging from 1∶30 to 2∶10^8^. The amplification rate is defined operationally as the highest dilution between PMCAb rounds at which amplification was still capable of compensating for the effect of dilution [34]. For 263K, the amplification rate increased approximately 50 fold, from 100-fold in NBH to 5000-fold in dsNBH (Figure 2B). For SSLOW, the amplification rate increased more than 5×10^5^ fold, from approximately 100-fold in NBH to at least 5×10^7^-fold in dsNBH (Figure 2B). Consistent with previous studies [32], [34], SSLOW showed an increase in signal intensity in serial PMCAb suggesting that it undergoes fast ‘adaptation’ to the PMCAb environment.

Both experimental formats showed that desialylation of PrP^C^ increases the rate of prion amplification in PMCAb, while the magnitude of an increase was strain-dependent. This effect was considerably higher for synthetic strains than strains of natural origin.

### Desialylation of PrP^C^ reduces or eliminates the cross-seeding barrier in PMCAb

**Figure 3:**
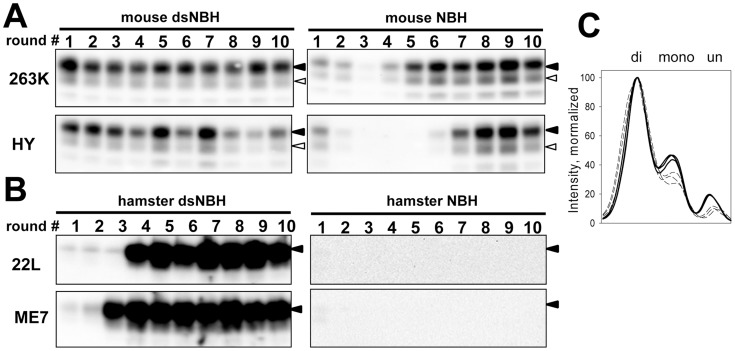
De-sialylation of PrP^C^ reduces species barrier in PMCAb. **A.** 263K or HY brain materials were diluted 10^3^-fold into 10% mouse dsNBH or NBH and subjected to ten serial PMCAb rounds with 5-fold dilution between rounds. Prior to electrophoresis, samples were treated with PK. Blots were stained with Ab3531 antibody. **B.** 22L or ME7 brain material was diluted 10^3^-fold into 10% hamster dsNBH or NBH and subjected to ten serial PMCAb rounds with 5-fold dilution between rounds. Prior to electrophoresis samples were treated with PK. Blots were stained with 3F4 antibody. Black and white triangles mark diglycosylated and monoglycosylated forms, respectively. **C.** PK-resistance profile illustrating relative representation of di-, mono-, and unglycosylated glycoforms in 263K-seeded PMCAb products produced in three reactions with mouse NBH (bold solid lines) or dsNBH (dashed lines).

In previous studies prion amplification in PMCA was shown to mimic key features of prion transmission including the species barrier [37]–[39]. A drop in amplification efficiency in PMCAb seeded with heterologous PrP^Sc^ will be referred to as the cross-seeding barrier. Considering that desialylation of PrP^C^ increases the rate of PrP^Sc^ amplification, we were interested to test whether desialylation of PrP^C^ eliminates the cross-seeding barrier in PMCAb. When serial PMCAb reactions in mouse NBH were seeded with hamster strains 263K or HY, stable replication was observed only after four or six serial rounds, respectively, a sign of a significant cross-seeding barrier (Figure 3A). However, when mouse dsNBH was used as a substrate for 263K and HY, stable amplification was observed starting from the first round for both strains with no signs of cross-seeding barrier (Figure 3A).

In a reverse transmission experiment, two mouse strains 22L and ME7 were subjected to amplification in hamster NBH. No signal was observed for ten serial rounds suggesting that these two strains could not cross the barrier under the current experimental conditions (Figure 3B). Surprisingly, when 22L and ME7 were subjected to amplification in hamster dsNBH, stable amplification was observed starting from the third or fourth serial round, respectively (Figure 3B). Both experiments show that reducing sialylation levels of PrP^C^ of a host species helps to eliminate or significantly reduce the barrier that prevents cross-seeding.

### The level of PrP^C^ sialylation controls PrP^Sc^ glycoform ratio

Careful comparison of mouse-adapted 263K or HY revealed that the relative ratio of diglycosylated vs. monoglycosylated glycoforms was higher in dsNBH-amplified products than in NBH-amplified products (Figure 3A,C). These changes suggest that the ratio of di-, mono- and unglycosylated glycoforms is not only a function of prion strain or host, but also depends on PrP^C^ sialylation status. Due to glycan sialylation, the surface of PrP^Sc^ particles has a dense negative charge that creates electrostatic repulsion and, presumably, limits the percentage of diglycosylated PrP^C^ able to be recruited by PrP^Sc^. We propose that desialylation of PrP^C^ eliminates electrostatic repulsion permitting a higher percentage of the diglycosylated glycoform to be accommodated.

**Figure 4:**
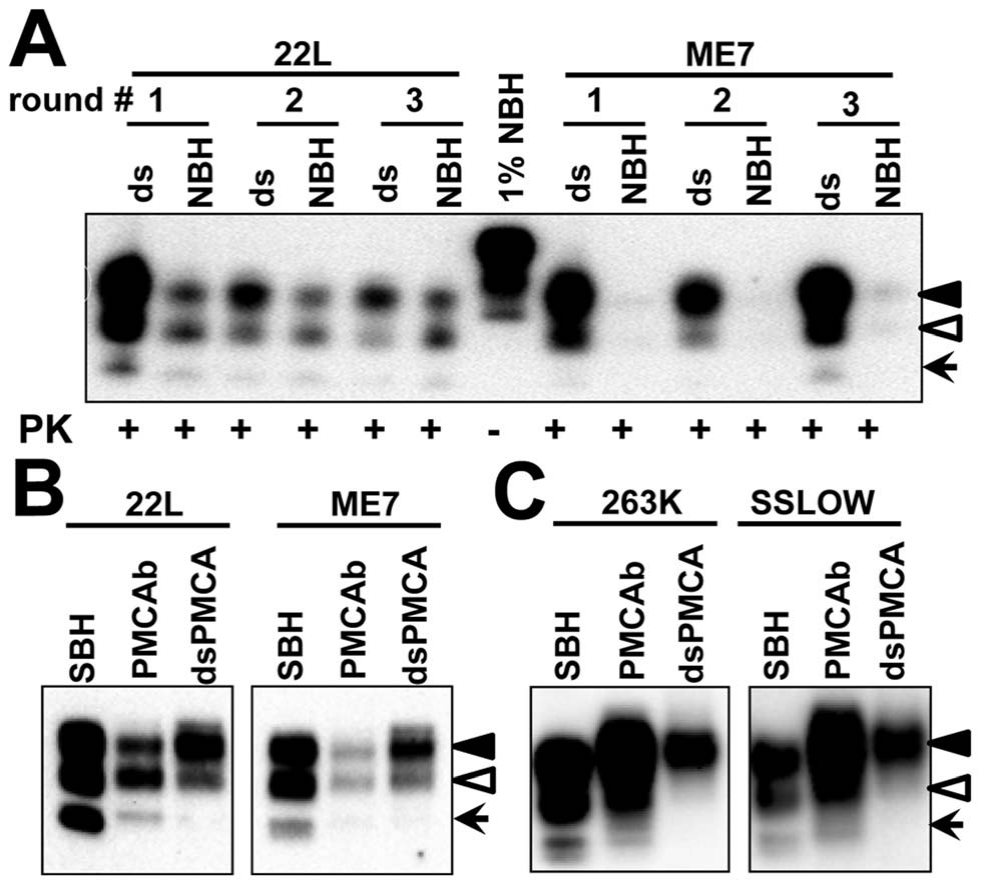
Influence of PrP^C^ sialylation level on glycoform distribution in PMCAb-derived products. **A.** 22L or ME7 brain material was diluted 5×10^2^- or 2×10^3^-fold, respectively, into 10% mouse dsNBH or NBH and subjected to three serial PMCAb rounds with 5-fold dilution between rounds. Prior to electrophoresis samples were treated with PK. Blot was stained with Ab3531 antibody. Undigested 1% mouse NBH was used as a reference. **B.** Western blot of 22L or ME7 scrapie brain material (SBH) or material produced in PMCAb using NBH or dsNBH (dsPMCAb), and stained with Ab3531 antibody. **C.** Western blot of 263K or SSLOW scrapie brain material (SBH) or material produced in PMCAb using NBH or dsNBH (dsPMCAb), and stained with 3F4 antibody. Black and white triangles mark di- and mono-glycosylated glycoforms, respectively, whereas arrows mark the unglycosylated form.

To test the hypothesis that PrP^C^ sialylation controls the ratio of glycoforms within PrP^Sc^, we examined the glycosylation profile of two mouse strains 22L and ME7 after their amplification in mouse NBH or dsNBH. Mouse strains were chosen because, in contrast to hamster strains, they have equal or even slightly higher percentage of monoglycosylated form relative to that of diglycosylated form. Upon amplification in dsNBH, the glycosylation profile of both 22L and ME7 changed immediately from predominantly monoglycosylated to predominantly diglycosylated (Figure 4A). For comparison, the glycosylation profile of NBH-amplified 22L PMCAb-derived material remained very similar to that of brain-derived 22L (Figure 4B). Due to very low amplification efficiency of ME7 in NBH (Figure 4A), it was difficult to compare the glycosylation profile of ME7 NBH- versus dsNBH-amplified material directly. Nevertheless, after adjusting the amount of material loaded on a gel, ME7 amplified in dsNBH showed a considerably higher ratio of di- versus mono- or unglycosylated glycoforms than those observed in ME7 NBH-amplified or brain-derived material (Figure 4B). Similar trend was observed for both hamster strains tested (263K and SSLOW). Monoglycosylated glycoform was well represented in brain-derived and in lower proportion in PMCAb-derived material, but absent in PMCAb-derived material produced in dsNBH (Figure 4C).

Taken together these results indicate that (i) the glycoform ratio within PrP^Sc^ is not only controlled by the strain or host but also by the sialylation status of PrP^C^; (ii) a decrease in PrP^C^ sialylation levels results in a higher percentage of diglycosylated glycoforms in PrP^Sc^; (iii) a shift toward diglycosylated glycoforms appears regardless of the host species, however the extent of the shift is likely determined by the strain-specific conformation.

### The sialylation status of PrP^C^ in brain and spleen is different

The lymphoreticular system plays an important role in prion pathogenesis, because: 1) prion replication in lymphoid tissues precedes neuroinvasion [40], [41], 2) lymphoid organs are targeted upon cross-species transmission and appear to be more permissive than central nervous system [42], and 3) inflammation facilitates prion invasion [43], [44]. Considering that desialylation of PrP^C^ facilitates PrP^Sc^ replication, we were interested in comparing the sialylation pattern of brain-derived and spleen-derived PrP^C^.

**Figure 5:**
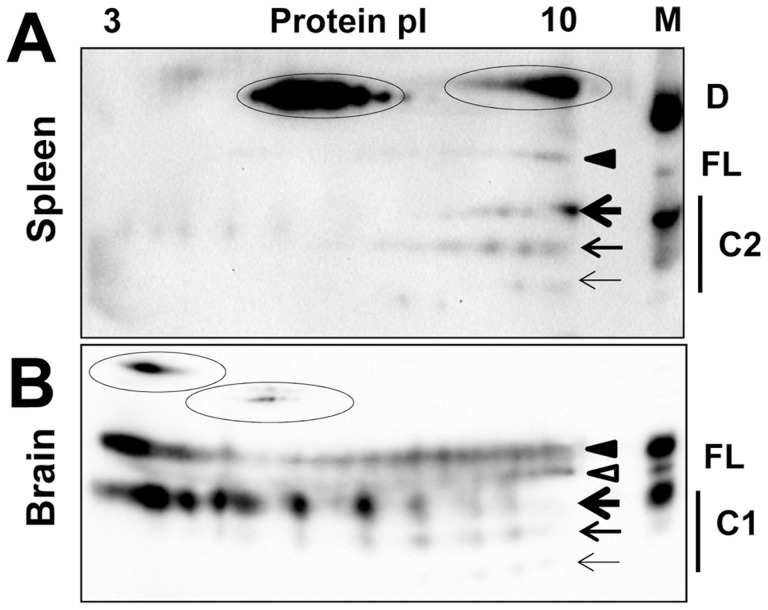
2D analysis of brain- and spleen-derived PrP^C^. 2D analysis of Syrian hamster spleen (**A**) and brain homogenates (**B**). Diglycosylated and monoglycosylated full-length PrP^C^ (FL) are marked by black and white triangles, respectively. Di-, mono- and unglycosylated C2 fragments (C2) in spleen or C1 fragments (C1) in brain are marked by bold, medium and thin arrows, respectively. PrP^C^, C1 or C2 dimers (D) are encircled. M stands for a marker lane: brain or spleen samples were diluted 10-fold and used as references for 2D gels. Blots were stained with SAF-84 antibody.

The 2D analysis of spleen tissues was very challenging because the level of PrP^C^ expression in the spleen is 20 to 50-fold lower than that in the brain. Moreover, most of spleen-derived PrP^C^ was proteolytically processed and formed a C2 fragment (residues ∼100–231) that was immunoreactive with 3F4 (epitope 109–112) and SAF-84 (epitope 160–170) antibodies, but not Ab3531 (epitope 90–102) antibody (Figure S1). This finding was in agreement with a previous report on N-terminal trimming of spleen-derived prion protein [45]. In addition, we found that the spleen-derived C2 fragment is highly prone to aggregation, as a significant proportion of it appeared as a dimer on SDS-gels (Figure S1). For the above reasons, 2D analysis of brain- and spleen-derived PrP^C^ was performed using the SAF-84 antibody that reacts with full-length PrP^C^ as well as C1 (residues 111–231) and C2 fragments (Figure 5).

Consistent with 1D SDS gel analysis, a significant percentage of full-length PrP^C^ and the C2 fragment were persistently seen as dimers on 2D gels of spleen tissues (Figure 5A). Pretreatment of spleen homogenates with sarcosyl did not help to reduce the amount of dimers. Nevertheless, a notable difference could be observed with respect to the charge distribution between spleen- and brain-derived monomeric full-length PrP^C^ (Figure 5A,B). In spleen material, the distributions of charge isoforms were shifted toward basic pH for both full-length PrP^C^ and the C2 fragment. While the details of sialylation of spleen-derived PrP^C^ remain to be investigated, this result suggests that the metabolism of PrP^C^ sialylation in spleen might be different from that in a brain.

### Correlation between PrP^Sc^ sialylation status and its infectivity

**Figure 6:**
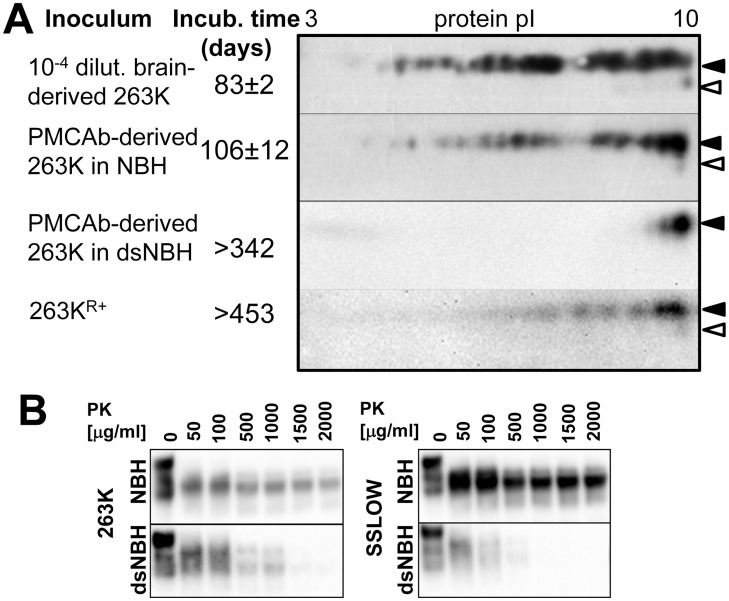
Correlation between PrP^Sc^ sialylation status and its infectivity. **A.** The following material was analyzed by 2D and animal bioassay: 1% brain-derived 263K material, PMCAb-derived 263K (products of 24th PMCAb rounds conducted in NBH with 10-fold dilution between rounds), PMCAb-derived 263K produced in dsNBH (products of 7^th^ PMCAb round conducted in dsNBH with 1000-fold dilution between rounds) and 263K^R+^ (263K brain material subjected to 12 serial PMCAb rounds in RNA-depleted NBH and then an additional 14 PMCAb rounds in NBH as described in [46]). Prior to inoculation, all PMCAb-derived materials were diluted 10-fold. 10^4^-fold diluted 263K brain material was used for inoculating a reference group to match the amount of PK-resistant material in PMCAb-derived samples. Diglycosylated and monoglycosylated PrP^C^ are marked by black and white triangles, respectively. **B.** Western blots of 263K- or SSLOW-seeded PMCAb-derived material produced using NBH or dsNBH and treated with increasing concentrations of PK as indicated. All blots were stained with 3F4 antibody.

To test whether PMCAb-associated changes in sialylation status affect prion infectivity, Syrian hamsters were inoculated with 263K brain-derived and PMCA-derived materials produced using NBH or dsNBH. We also attempted to produce desialylated PrP^Sc^ directly by treating brain-derived 263K with sialidase. However, this approach was not successful. For PMCAb conducted in NBH, 10^3^-fold diluted 263K brain material was subjected to 24 serial rounds with 1∶10 dilution between rounds that resulted in a final dilution of brain material of 10^−27^. For PMCAb conducted in dsNBH, 10^5^-fold diluted 263K brain material was subjected to 7 serial rounds with 1∶1000 dilutions between rounds that resulted in a final dilution of brain material of 10^−26^. On 2D gels, the charge distribution of products generated in PMCAb with dsNBH showed substantially more significant shift toward pI 10 and very little charge heterogeneity in comparison to the brain-derived or PMCAb-derived materials (Figure 6A).

The incubation time to the clinical signs of disease was 83±2 and 106±12 days in groups inoculated with brain-derived 263K and PMCAb-derived material, respectively. Two animals inoculated with PMCAb-derived material produced in dsNBH were sacrificed at 223 days p.i., and no PK-resistant material was found in their brains (Figure S2A). The remaining four animals from this group did not show any clinical signs and were sacrificed at 341 days p.i. No PK-resistant products were detected in their brain using 3F4 or SAF-84 antibodies (Figure S2B). This result suggests that in contrast to the material produced in PMCAb with NBH, the material produced in PMCAb with dsNBH had no detectible infectivity despite being subjected to fewer PMCAb rounds.

Lack of infectivity of PMCAb material produced in dsNBH could be due to changes in physical properties, particularly the high sensitivity to proteolytic clearance. To examine proteolytic resistance, PMCAb products generated in NBH or dsNBH were treated with increasing concentrations of PK (Figure 6B). Indeed, for both strains 263K and SSLOW, the products formed using desialylated substrate were substantially less resistant than PMCAb products produced in NBH (Figure 6B). Increased proteolytic sensitivity could lead to faster clearance of such inocula.

In previous studies, we showed that 263K gave rise to a novel PrP^Sc^ conformation referred to as 263K^R+^ after 263K brain material was subjected to 12 serial PMCAb rounds in RNA-depleted NBH and then to additional 14 PMCAb rounds in NBH [46]. While 263K^R+^ amplified very fast *in vitro*, no clinical disease was observed upon inoculation of 263K^R+^ in first and second serial passages. We were interested in testing whether lack of infectivity could be due to changes in sialylation status of 263K^R+^. 2D analysis revealed that the distribution of 263K^R+^ charge isoforms was considerably shifted to the basic pI in comparison to that of PMCAb-derived 263K (Figure 6A). Therefore, for both 263K^R+^ and PMCAb-derived material produced in dsNBH the lack of infectivity correlates well with their low sialylation status.

### Sialidase 1 (Neu1) deficiency does not affect the sialylation status of PrP^C^

The catabolism of sialoglycoconjugates is regulated by four sialidases (also referred to as neuraminidases Neu1, Neu2, Neu3 and Neu4), all of which catalyze the removal of terminal sialic acid residues from carbohydrates of glycoprotein or glycolipids [47]. While all four sialidases are expressed in neuronal tissues, their levels and subcellular localization differ greatly. Because Neu1 is the most abundant, is expressed in the brain and is localized in lysosomes and on the cell surface, we decided to examine its role in regulating PrP^C^ sialylation status. Brain materials from *Neu1^−/−^*, *Neu1^+/−^* and wild type mice of two genetic backgrounds (FVB and BL6) were compared using 2D analysis. No notable differences with respect to PrP^C^ charge distribution were observed between wild type, *Neu1^−/−^* or *Neu1^+/−^* mice of the two groups (Figure S3). The lack of effect could be because (i) Neu1 is not involved in PrP^C^ desialylation, (ii) Neu1 deficiency is compensated by other neuraminidases, or (iii) PrP^C^ molecules are degraded very fast in lysosomes, so the relative contribution of desialylated PrP^C^ in the total pool of PrP^C^ is very small. To probe the possible role of Neu1 in PrP catabolism further, we also examined the sialylation profile of PrP proteolytic fragment C1 (residues ∼111–231) using SAF-84 antibody. C1 is present in large amounts in mouse brain and similar to PrP^C^, C1 can be found in di-, mono- and unglycosylated forms; however, the dynamics of the cellular clearance of C1 and its cellular localization could be different from those of full-length PrP^C^. No differences in sialylation status of C1 fragments were found between *Neu1^−/−^*, *Neu1^+/−^* and wild type mice (Figure S3). Because the life-span of *Neu1^−/−^* mice is limited to ∼150 days, it was not possible to test whether knocking out *Neu1* affected the incubation time to prion disease.

## Discussion

Sialic acids are the most abundant terminal monosaccharides in cell membrane glycans [21], [22]. Sialylation plays an essential role in key cellular functions including cell signaling, adhesion, differentiation, neuronal plasticity, cell-cell and cell-pathogen recognition, and the activation and trafficking of B and T lymphocytes, among other things [21], [22]. The sialic acid content is the highest in embryonic and perinatal phases, but drops gradually during adulthood [48], [49]. Brain and immune tissues including spleen have considerably higher amounts of sialic acid in their membrane fraction than other organs such as heart or kidney [48].

**Figure 7:**
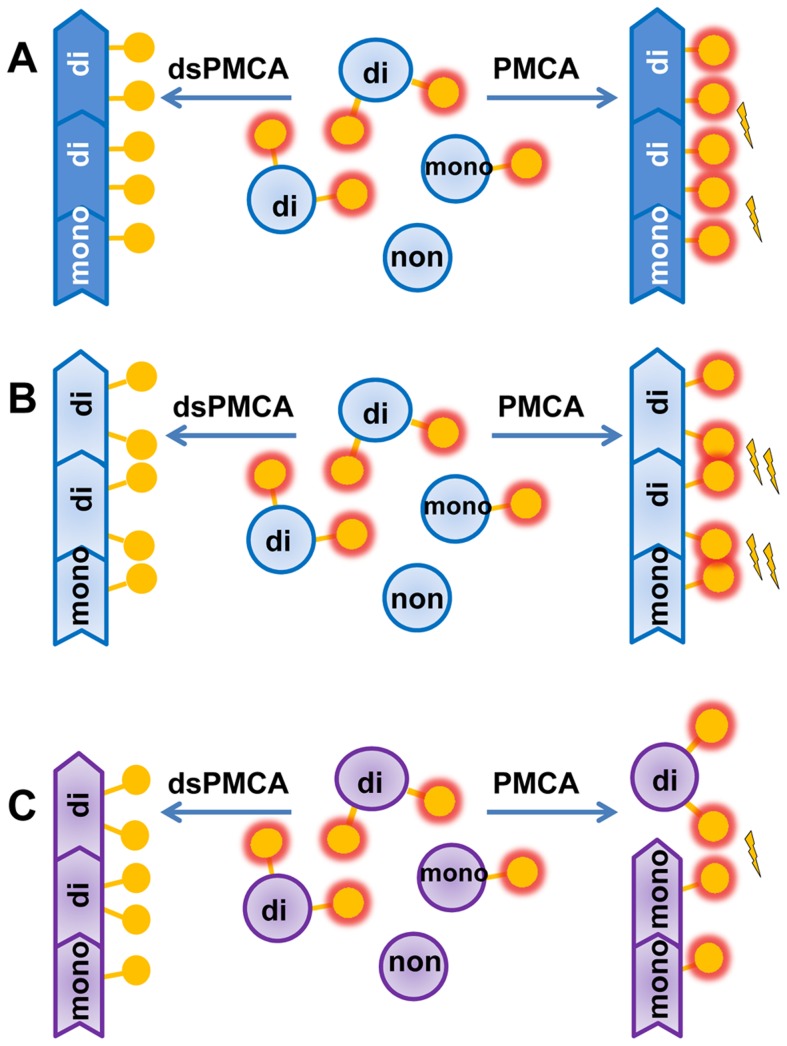
Schematic diagrams illustrating that prion polymerization and PrP^Sc^ glycoform ratio are controlled by sialic acid residues of glycans. **A, B.** Sialylation of glycans impedes prion amplification in PMCAb due to electrostatic repulsion between sialic acid residues on the PrP^Sc^ surface. Desialylation of a substrate facilitates prion polymerization in dsPMCAb. Due to strain-specific differences in PrP^Sc^ structures, PrP^C^ desialylation speeds up polymerization of strains of synthetic origin (**B**) much stronger than strains of natural origin (**A**). **C.** Electrostatic repulsion between sialic acid residues limits percentage of diglycosylated glycoforms that can be accommodated in PrP^Sc^. Desialylation of PrP^C^ in dsPMCAb changes the ratio of glycoforms in PrP^Sc^ in favor of diglycosylated glycoforms. PrP^C^ monomers are depicted as blue circles; glycosyls are shown as yellow circles where presence of terminal sialic acid residues is shown as a red glow. Electrostatic repulsion is represented with lightning bolts.

In the current study we showed that de-sialylation of PrP^C^ increases PrP^Sc^ amplification rates in PMCAb. Faster amplification of desialylated substrates was likely due to removal of electrostatic repulsion between glycan moieties, which can carry up to 4 negatively charged sialic acid residues each (Fig. 7A,B). This work suggests that a dense negative charge on the surface of PrP^Sc^ particles due to sialylated glycans prevents efficient PrP^Sc^ replication. Previous studies revealed that partial removal of N-linked glycans from PrP^C^ using treatment with PNGase F or replacing a mixture of di-, mono- and unglicosylated PrP^C^ with only unglicosylated form have a negative effect on PrP^Sc^ amplification in PMCA [50], [51]. Taken together, these results indicate that while glycans are important for efficient amplification of PrP^Sc^, the terminal sialic acid residues have a negative impact.

Notably, the positive effect of PrP^C^ desialylation on the replication rate, while present in all prion strains probed in this study, differed considerably in magnitude. Approximately 10–50 fold increases in the rate for strains of natural origin (263K, HY, Drowsy) was strikingly different from the 10,000-fold increase for the strains of synthetic origin. Such drastic difference between strains of the two classes indicate that steric clashes between sialic acid residues in neighboring glycans are much more substantial in synthetic strains than in strains of natural origin (Figure 7A,B). The elimination of the negative charges from PrP^Sc^ surface led to a much more significant drop in polymerization energy costs for synthetic strains than natural strains. This result highlights structural differences between the two classes of prion strains.

The hypothesis that electrostatic repulsion between sialic residues controls PrP^Sc^ amplification rate explains why undersialylated PrP^C^ molecules are preferentially recruited during *in vitro* amplification at the expense of oversialylated PrP^C^. As a result, the sialylation status of PrP^Sc^ changes during PMCAb becoming less sialylated in comparison to brain-derived PrP^Sc^. In previous studies, prion specific infectivity (the ratio of the infectivity titer to the amount of PrP^Sc^) was found to decrease gradually during amplification in serial PMCA [52]. A decrease in specific infectivity correlates well with a drop in sialylation status of PMCAb-derive material observed here. In support of this correlation, the current study observed longer incubation time to disease for PMCAb-derived material relative to that of brain-derived PrP^Sc^, and a lack of clinical signs for PMCAb-derived material produced using desialylated substrate. Recent studies reported a gradual change in strain-specific secondary structure during serial amplification in PMCAb [33]. The relationship between changes in PrP^Sc^ conformation and sialylation status during serial PMCAb is not clear. Nevertheless, the fact that PMCAb material produced in NBH caused prion disease after 24 PMCAb rounds, whereas PMCAb material amplified in dsNBH did not cause the disease after only 7 amplification rounds, suggests that it is the loss of sialylation rather than a number of PMCAb rounds that had deleterious effect on infectivity.

Progression of prion diseases is determined by a number of factors including PrP^C^-to-PrP^Sc^ conversion rate, PrP^Sc^ clearance, PrP^Sc^ deposition sites, and relative toxicity and size of PrP^Sc^ aggregates. Lack of clinical disease in animals inoculated with desialylated PMCAb products can be attributed in part to their high clearance rate. Consistent with this hypothesis, PMCAb materials produced with de-sialylated substrate were more sensitive to proteolytic digestion than standard PMCAb-derived material. Alternative mechanisms that involve interaction with microglia and cells of the immune system might also contribute to the lack of infectivity of PMCAb material produced in dsNBH. The mammalian immune system uses terminal sialylation of cell surface glycoproteins to identify pathogenic microorganisms to set them apart from their own cells, as microorganisms generally lack enzymes essential for sialic acid synthesis [21], [22]. In the absence of terminal sialic residues, galactose is exposed as the terminal residue of glycans of microbial glycoproteins and serves as a signal for activating the immune response and phagocytotic clearance by macrophages [21], [22]. If clearance of PrP^Sc^ involves mechanisms that are involved in clearance of microorganisms, desialylated PrP^Sc^ should be cleared much faster than sialylated PrP^Sc^. Notably, some microbial pathogens recruit sialic acid from the host and sialylate their own glycoproteins in order to become “invisible” to the host's immune systems [23]. In addition to the clearance by macrophages, recent studies revealed that glycoclusters with terminal sialic acid were stable upon injection into mice and accumulated in the spleen, while the same clusters without sialic acid residues were rapidly excreted via the urinary tract [53]. It remains to be determined whether any of the above mechanisms account for lack of infectivity of desialylated PMCAb material. Nevertheless, fast clearance of PrP^Sc^ with low level of sialylation in a brain and luck of such clearance in PMCAb could explain why the sialylation levels of brain-derived and PMCAb-derived PrP^Sc^ are different.

In previous studies, prions with high infectivity titers that lacked sialylation were generated *in vitro* using recombinant PrP [54], [55]. Because entire carbohydrate groups were missing in PrP^Sc^ produced from recombinant PrP, it is unlikely that the immune system and microglia can identify these synthetic prions as potential pathogens in the same manner as it deals with desialylated PMCAb products. Consistent with this hypothesis, scrapie brain material from transgenic mice deficient in PrP glycosylation at both sites was found to be capable of infecting wild type mice [56]. Notably, transgenic mice that lacked glycosyls at both sites displayed a dramatic increase in the incubation time, incomplete attack rate or lack of infection [56]. These results suggest that in the absence of glycosylation/sialylation, PrP^C^ of the host does not support well the infection or the newly formed PrP^Sc^ is not toxic.

That PrP^C^ sialylation controls PrP^C^-to-PrP^Sc^ conversion rate has far reaching implications. A decrease in PrP^C^ sialylation could lead to a dramatic plunge of PrP^C^-to-PrP^Sc^ barriers *in vivo* and provide favorable conditions for (i) lowering the energy barrier of the spontaneous PrP^C^-to-PrP^Sc^ conversion in sporadic prion diseases; (ii) successful infection of a host or tissues with abnormally low sialylation status by low prion doses; and (iii) crossing the species barrier. In support of the last hypothesis, the current study revealed that hamster-to-mouse or mouse-to-hamster cross-seeding barriers can be reduced or abolished entirely in PMCAb, if de-sialylated PrP^C^ is used as a substrate. While PMCAb can not predict the outcomes of transmission species barrier effects in whole organisms, our work opens an intriguing possibility that a species barrier is not only controlled by PrP amino acid sequence and PrP^Sc^ strain-specific structure but also by PrP^C^ sialylation status. Noteworthy, because of an irreversible mutation in the gene encoding human N-acetylneuraminic acid hydroxylase, humans and the rest of mammalian species use different sialyc acid residues: humans produce only N-acetyl neuraminic acid (Neu5Ac), while other mammals produce Neu5Ac and N-glycolylneuraminic acid (Neu5Gc) [57]. The difference in sialic acid structure affects interaction of pathogenic microbes with the immune systems of humans and other mammalian species. This difference might also contribute to a previously unappreciated mechanism that controls the prion transmission barrier between mammals and humans.

Lymphoid organs are targeted upon cross-species transmission and appear to be more permissive than central nervous system [42]. 2D analysis of PrP^C^ charge distribution revealed that spleen-derived PrP^C^ was different from that of brain-derived PrP^C^ with respect to their sialylation pattern, although precise comparison of the two tissues was complicated because of the low expression level, high tendency for aggregation and formation of the C2 proteolytic fragment by spleen-derived PrP^C^. Further research is needed more sensitive and accurate tools to confirm whether hyposialylation of PrP^C^ in spleen makes the spleen more susceptible to prion infection than brain. Noteworthy, endogenous sialidase activity was found to increase in cells of the immune system, including lymphocytes and monocytes during cell activation and differentiation leading to undersialylation of cell surface glycoproteins [58], [59]. Because inflammatory conditions support prion replication [43], [44], it would be interesting to examine in future studies whether inflammation-induced activation of sialidase gives rise to undersialylated PrP^C^ and facilitates prion infection.

Sialylation status of glycoproteins is controlled by sialyltransferases and sialidases (also called neuraminidases), two classes of enzymes that transfer or cleave terminal sialic acids to/from glycoproteins, respectively [60]. In mammals, there are four sialidases (Neu1, Neu2, Neu3 and Neu4) that are expressed in a tissue-dependent manner and differ with respect to their cellular localization and enzymatic properties [60]. Among the four sialidases, Neu1 is the most abundant and ubiquitously distributed. It is a part of a multi-enzyme, 1200 kDa hydrolase complex, which is localized predominantly in lysosomes and to lesser extent on the cell surfaces of many tissues and organs including brain [61]. To test whether Neu1 is responsible for desialylation of PrP^C^, brain materials from *Neu1^-/-^* knockout and *Neu1^+/−^* mice generated in two genetic backgrounds (FVB and BL6) were analyzed using 2D gels. No significant changes in PrP^C^ sialylation patterns were observed in *Neu1^−/−^* or *Neu1^+/−^* in comparison to those of corresponding wild type mice (Figure S1). These results indicate that either Neu1 is not involved in PrP^C^ desialylation or Neu1 deficiency is compensated by other neuraminidases. Alternatively, PrP^C^ molecules might be degraded so fast following desialylation, that the relative contribution of desialylated PrP^C^ in the total pool of PrP^C^ is too small to be detected by the current approach. In either case, Neu1 might not be the right target if one wants to alter the sialylation status of PrP^C^ *in vivo* for therapeutic intervention.

The ratio of di-, mono- and unglycosylated glycoforms within PrP^Sc^ is believed to be an intrinsic property of a prion strain or PrP^Sc^ subtype (in sporadic prion diseases) [62]–[65]. As such, the PrP^Sc^ glycoform ratio is used widely for strain typing and classification of PrP^Sc^ subtypes in sporadic CJD [64], [65], and changes in the glycoform ratio are thought to be indicative of a strain mutation or strain adaptation to a new host or environment [66], [67]. Surprisingly, the current study revealed that the PrP^Sc^ glycoform ratio is not only controlled by prion strain or the host, but also by the sialylation status of PrP^C^. A decrease in PrP^C^ sialylation levels resulted in a shift of the glycoform ratio toward diglycosylated forms at the expenses of mono- and unglycosylated glycoforms for both mouse and hamster strains. Such a relationship is explained well by the model that postulates that electrostatic repulsion created by sialic acid residues on the surface of PrP^Sc^ particles limits the percentage of diglycosylated molecules that can be accommodated within PrP^Sc^ (Figure 7C). A decrease in sialylation levels reduces electrostatic repulsion leading to an increase in the percentage of diglycosylated molecules. In previous studies, treatment of prion-infected cultured cells with swainsonine, a compound that blocks synthesis of complex N-linked glycans, was shown to select minor strain variants or “mutants” resistant to swainsonine [68]. This process was accompanied by a change in the PrP^Sc^ glycoform ratio in favor of diglicosylated forms at the expense of monoglycosylated PrP^Sc^ glycoforms [68], [69]. The extent to which swainsonine-related selection of minor variants and changes in glycoform ratio were due to lack of sialic acid residues is unclear.

Recent studies established a possible link between protein sialylation and Alzheimer's diseases [70]. Deficiency of the lysosomal sialidase Neu1 was found to lead to the spontaneous occurrence of an Alzheimer's disease-like amyloidogenic process in mice. Loss of Neu1 resulted in accumulation of an over-sialylated amyloid precursor protein in lysosomes and excessive release of Aβ peptides by lysosomal exocytosis [70].

The current study opens a new avenue in prion research that might shed new light on the mechanism of prion replication and contribute to development of new therapeutic approaches. A number of sialic acid metabolic precursors or sialidase inhibitors are currently available and approved by FDA. Nevertheless, the impact and effectiveness of pharmacological intervention that target PrP^C^ sialylation on progression of prion diseases are difficult to predict. The sialylation status of PrP^C^ does not only control the rate of prion replication and magnitude of the species barrier, but is also likely to affect prion uptake and transport by macrophages, prion clearance rate, toxicity of PrP^Sc^ particles, and interaction of PrP^Sc^ with cells of the immune system and microglia. These topics have to be investigated in future studies.

In summary, the current work demonstrated that hyposialylated PrP^C^ molecules are a preferable substrate for prion amplification in PMCAb. PMCAb-derived PrP^Sc^ is less sialylated than brain-derived PrP^Sc^. De-sialylation of PrP^C^ significantly speeds up PrP^Sc^ amplification in a strain-dependent manner and significantly reduces or eliminates the species barrier. A decrease in PrP^Sc^ sialylation correlates with a drop in infectivity of PMCAb-derived material. The sialylation status of brain-derived PrP^C^ appears to differ from that of spleen-derived PrP^C^. The sialylation status of PrP^C^ controls the PrP^Sc^ glycoform ratio with a decrease in PrP^C^ sialylation levels resulting in a higher percentage of the diglycosylated glycoform in PrP^Sc^. Knocking out lysosomal sialidase Neu1 does not change the sialylation status of PrP^C^. The current work highlights the previously unappreciated role of PrP^C^ sialylation in prion diseases and opens new directions in prion research, including development of new therapeutic approaches.

## Materials and Methods

### Ethics statement

This study was carried out in strict accordance with the recommendations in the Guide for the Care and Use of Laboratory Animals of the National Institutes of Health. The protocol was approved by the Institutional Animal Care and Use Committee of the University of Maryland, Baltimore (Assurance Number A32000-01; Permit Number: 0309001).

### Normal and scrapie brain material

Hyper and Drowsy scrapie brain materials were kindly provided by Richard Bessen (Colorado State University, Fort Collins, CO); 263K, 22L and ME7 scrapie brain materials were kindly provided by Robert Rohwer (Veterans Affair Maryland Health Care System, Baltimore, MD); SSLOW scrapie brain homogenate was prepared using animals from the 4th passage of SSLOW [71]. *Neu1^−/−^*, *Neu1^+/−^* and wild type mouse brains were collected from four month old FVB mice and five month old BL6 mice [72]. The mice were perfused with 20 ml PBS/5mM EDTA (pH 7.4), and then brains were collected and frozen in liquid nitrogen.

### Bioassay

Weanling Golden Syrian hamsters (all males) were inoculated intracerebrally under 2% O_2_/4 MAC isoflurane anesthesia. Each animal received 50 µl of brain homogenate or PMCAb products. After inoculation, hamsters were observed daily for disease using a ‘blind’ scoring protocol. Hamsters were euthanized as they approach the terminal stage of the disease.

### Protein misfolding cyclic amplification with beads (PMCAb)

10% normal brain homogenate (NBH) from healthy hamsters was prepared as described previously [35] and used as a substrate for PMCAb [39]. The sonication program consisted of 20 sec sonication pulses delivered at 170W energy output applied every 20 min during a 24 hour period. For each subsequent round, 10 or 20 µl of the reaction from the previous round were added to 90 or 80 µl of fresh substrate, respectively. Each PMCAb reaction was carried out in the presence of two 2/32” Teflon beads (AmazonSupply.com).

To analyze production of PK-resistant PrP material in PMCAb, 10 µl of sample were supplemented with 5 µl SDS and 5 µl PK, to a final concentration of 0.25% SDS and 50 µg/ml PK, followed by incubation at 37°C for 1 hour. The digestion was terminated by addition of SDS sample buffer and heating the samples for 10 min in a boiling water bath. Samples were loaded onto NuPAGE 12% BisTris gels, transferred to PVDF membrane, and probed with SAF-84, Ab3531 or 3F4 antibodies.

### Treatment of NBH with *A.ureafaciens* sialidase

To produce de-sialylated substrate, 10% NBH from healthy hamsters prepared for PMCAb was treated with *Arthrobacter ureafaciens* sialidase (cat # N3786, Sigma-Aldrich, St. Louis, MO) as follows. The lyophilized enzyme was dissolved in MilliQ water to the final concentration of 500mIU/ml. After preclearance of NBH at 500 × *g* for 2 min and addition of the buffer supplied by manufacturer, 7mIU/ml sialidase were added to the supernatant, and the reaction was incubated on a rotator at 37 °C for 5 h. The resulting substrate was used in dsPMCAb using the sonication protocol described for PMCAb. To prepare mock sialidase treated PMCAb substrate, the procedures were the same with adding MilliQ water instead of sialidase solution.

### 2D electrophoresis

Samples of 10 µl volume prepared in 1xSDS sample loading buffer as described above were solubilized for 1h at room temperature in 80 µl solubilization buffer (8M Urea, 2% CHAPS, 5mM TBP, 20mM Tris pH 8.0), alkylated by addition of 135 µl 0.5M iodoacetamide and incubated for 1h at room temperature. Then, 1150 µl of ice-cold methanol was added, and samples were incubated for 2h at −20°C. After centrifugation at 13,000 rpm and 4°C, supernatant was discarded and the pellet was re-solubilized in 160 µl rehydration buffer (7M urea, 2 M thiourea, 1%DTT, 1% CHAPS, 1% Triton X-100, 1% ampholyte, trace amount of Bromophenol Blue). Fixed immobilized pre-cast IPG strips (cat. # ZM0011, Life Technologies, Carlsbad, CA) with a non-linear pH gradient 3–10 were rehydrated in 155 µl of resulting mixture overnight at room temperature inside IPGRunner cassettes (cat. # ZM0008, Life Technologies, Carlsbad, CA). Isoelectrofocusing (first dimension separation) was performed at room temperature with rising voltage (175V for 15 minutes; 175–2,000V linear gradient for 45 minutes; 2,000V for 30 minutes) on Life Technologies Zoom Dual Power Supply using the XCell SureLock Mini-Cell Electrophoresis System (cat. # EI0001, Life Technologies). The IPG strips were then equilibrated for 15 minutes consecutively in (i) 6 M Urea, 20% glycerol, 2% SDS, 375mM Tris-HCl pH 8.8, 130mM DTT, and (ii) 6 M Urea, 20% glycerol, 2% SDS, 375mM Tris-HCl pH 8.8, 135mM iodoacetamide, and loaded on 4–12% Bis-Tris ZOOM SDS-PAGE pre-cast gels (cat. # NP0330BOX, Life Technologies). For the second dimension, SDS-PAGE was performed for 1h at 170V. Immunoblotting was performed as described above.

## Supporting Information

Figure S1 **Western blot analysis of spleen- and brain-derived PrP^C^.** Spleen- and brain-derived PrP^C^ from non-infected animals was analyzed using Western blotting. The vast majority of spleen-derived PrP^C^ appeared as a C2 proteolytic fragment at 25 kDa with its dimmer at 50 kDa. Blots were stained with Ab3531, 3F4 or SAF-84 antibodies as indicated.
(TIF)

Figure S2 **Western blot analysis of brain material from animals inoculated with de-sialylated PMCAb products.** **A.** Animals #1 and 2 (lanes 3 and 4) were inoculated with PMCAb-derived materials produced using dsNBH. No clinical signs were developed and animal were euthanized at 223 days p.i. Initial inoculum is shown in lane 5; 1% 263K brain homogenate is shown as a reference in lane 2. **B.** Animals #1 to 6 were inoculated with PMCAb-derived materials produced using dsNBH and euthanized at 223 or 342 days p.i. Non-digested 1% NBH and PK-treated 1% 263K brain homogenates are shown as references. 3F4 or SAF-84 antibodies were used for staining.
(TIF)

Figure S3 **2D analysis of PrP^C^ and C1 in wild type,** ***Neu1^−/−^*** **and** ***Neu1^+/−^*** **mice.** 2D analysis of 10% brain homogenates from wild type, *Neu1^−/−^* or *Neu1^+/−^* mice of FVB or BL6 genetic background. Blots were stained with Ab3531 or SAF-84 antibody, as indicated. M stands for a marker lane: brain samples were diluted 10-fold and used as references for 2D gels.
(TIF)

## References

[1] Prusiner SB (1998) Prions. Proc Natl Acad Sci U S A 95: 13363–13383. 9811807 10.1073/pnas.95.23.13363 PMC33918

[2] Cohen FE, Prusiner SB (1998) Pathologic conformations of prion proteins. Annu Rev Biochem 67: 793–819. 9759504 10.1146/annurev.biochem.67.1.793

[3] Stahl N, Baldwin MA, Hecker R, Pan KM, Burlingame AL, et al (1992) Glycosylinositol phospholipid anchors of the scrapie and cellular prion proteins contain sialic acid. Biochemistry 31: 5043–5053. 1350920 10.1021/bi00136a600

[4] Stahl N, Borchelt DR, Hsiao K, Prusiner SB (1987) Scrapie prion protein contains a phosphatidylinositol glycolipid. Cell 51: 229–240. 2444340 10.1016/0092-8674(87)90150-4

[5] Turk E, Teplow DB, Hood LE, Prusiner SB (1988) Purification and properties of the cellular and scrapie hamster prion proteins. Eur J Biochem 176: 21–30. 3138115 10.1111/j.1432-1033.1988.tb14246.x

[6] Bolton DC, Meyer RK, Prusiner SB (1985) Scrapie PrP 27–30 is a sialoglycoprotein. J Virol 53: 596–606. 3918176 10.1128/jvi.53.2.596-606.1985 PMC254675

[7] Stahl N, Baldwin MA, Teplow DB, Hood L, Gibson BW, et al (1993) Structural studies of the scrapie prion protein using mass spectrometry and amino acid sequencing. Biochemistry 32: 1991–2002. 8448158 10.1021/bi00059a016

[8] Endo T, Groth D, Prusiner SB, Kobata A (1989) Diversity of oligosaccharide structures linked to asparagines of the scrapie prion protein. Biochemistry 28: 8380–8388. 2574992 10.1021/bi00447a017

[9] Rudd PM, Endo T, Colominas C, Groth D, Wheeler SF, et al (1999) Glycosylation differences between the normal and pathogenic prion protein isoforms. Proc Natl Acad Sci U S A 96: 13044–13049. 10557270 10.1073/pnas.96.23.13044 PMC23897

[10] DeArmond SJ, Qiu Y, S nchez H, Spilman PR, Ninchak-Casey A, et al (1999) PrPC glycoform heterogeneity as a function of brain region: implications for selective targeting of neurons by prion strains. J Neuropathol Exp Neurol 58: 1000–1009. 10499442 10.1097/00005072-199909000-00010

[11] Pan T, Colucci M, Wong BS, Li R, Liu T, et al (2001) Novel Differences between Two Human Prion Strains Revealed by Two-dimensional Gel Electrophoresis. J Biol Chem 276: 37284–37288. 11489910 10.1074/jbc.M107358200

[12] Yuan J, Xiao X, McGeehan J, Dong Z, Cali I, et al (2006) Insoluble Aggregates and Protease-resistant Conformers of Prion Protein in Uninfected Human Brains. J Biol Chem 281: 34848–34858. 16987816 10.1074/jbc.M602238200

[13] Zanusso G, Farinazzo A, Prelli F, Fiorini F, Gelati M, et al (2004) Identification of distinct N-terminal truncated forms of prion protein in different Creutzfeldt-Jakob disease subtypes. J Biol Chem 279: 38936–38942. 15247220 10.1074/jbc.M405468200

[14] Pan T, Li A, Wong BS, Liu T, Gambetti P, et al (2002) Heterogeneity of normal prion protein in two- dimensional immunoblot: presence of various glycosylated and truncated forms. J Neurochem 81: 1092–1101. 12065622 10.1046/j.1471-4159.2002.00909.x

[15] Schmitz M, Lullmann K, Zafar S, Ebert E, Wohlhage M, et al (2014) Association of prion protein genotype and scrapie prion protein type with cellular prion protein charge isoform profiles in cerebrospinal fluid of humans with sporadic or familial prion diseases. Neurobiol Aging 35: 1177–1188. 24360565 10.1016/j.neurobiolaging.2013.11.010

[16] Stimson E, Hope J, Chong A, Burlingame AL (1999) Site-specific characterization of the N-linked glycans of murine prion protein by high-performance liquid chromatography/electrospray mass spectrometry and exoglycosidase digestions. Biochemistry 38: 4885–4895. 10200178 10.1021/bi982330q

[17] Monnet C, Marthiens V, Enslen H, Frobert Y, Sobel A, et al (2003) Heterogeneity and regulation of cellular prion protein glycoforms in neuronal cell lines. Eur J Neurosci 18: 542–548. 12911750 10.1046/j.1460-9568.2003.02777.x

[18] Novitskaya V, Makarava N, Sylvester I, Bronstein IB, Baskakov IV (2007) Amyloid fibrils of mammalian prion protein induce axonal degeneration in NTERA2-derived terminally differentiated neurons. J Neurochem 102: 398–407. 17472702 10.1111/j.1471-4159.2007.04537.x

[19] Beringue V, Mallinson G, Kaisar M, Tayebi M, Sattar Z, et al (2003) Regional heterogeneity of cellular prion protein isoforms in the mouse brain. Brain 126: 2065–2073. 12821516 10.1093/brain/awg205

[20] Goh AXH, Li C, Sy MS, Wong BS (2007) Altered prion protein glycosylation in the aging brain. J Neurochem 100: 841–854. 17144900 10.1111/j.1471-4159.2006.04268.x

[21] Varki A, Gagneux P (2012) Multifarious roles of sialic acid in immunity. Annals of the New York Academy of Sciences 1253: 16–36. 22524423 10.1111/j.1749-6632.2012.06517.x PMC3357316

[22] Marth JD, Grewal PK (2008) Mammalian glycosylation in immunity. Nat Rev Immunology 8: 874–887. 18846099 10.1038/nri2417 PMC2768770

[23] Kooyk Y, Rabinovich GA (2008) Protein-glycan interactions in the control of innate and adaptive immune responses. Nat Immunology 9: 593–601. 18490910 10.1038/ni.f.203

[24] Li C, Wong P, Pan T, Xiao F, Yin S, et al (2007) Normal cellular prion protein is a ligand of selectins: binding requires Lex but is inhibited by sLex. Biochem J 406: 333–341. 17497959 10.1042/BJ20061857 PMC1948967

[25] Bradford BM, Crocker PR, Mabbott NA (2014) Peripheral prion disease pathogenesis is unaltered in the absence of sialoadhesin (Siglec-1/CD169). Immunology 143: 120–129. 24684244 10.1111/imm.12294 PMC4137961

[26] Bate C, Williams A (2012) Neurodegeneration Induced by Clustering of Sialylated Glycosylphosphatidylinositols of Prion Proteins. J Biol Chem 287: 7935–7944. 22262833 10.1074/jbc.M111.275743 PMC3318732

[27] Bate C, Williams A (2012) Clustring of sialylated glycocylphosphatidylinositol anchors mediated PrP-induced activation of cytoplasmic phospholipase A2 and synapse damage. Prion 6: 350–353. 22895089 10.4161/pri.21751 PMC3609062

[28] Makarava N, Kovacs GG, Bocharova OV, Savtchenko R, Alexeeva I, et al (2010) Recombinant prion protein induces a new transmissible prion disease in wild type animals. Acta Neuropathol 119: 177–187. 20052481 10.1007/s00401-009-0633-x PMC2808531

[29] Qin K, Yang Y, Mastrangelo P, Westaway D (2002) Mapping Cu(II) binding sites in prion proteins by diethyl pyrocarbonate modification and matrix assisted laser desorption ionization-time of flight (MALDI-TOF) mass spectrometric footprinting. J Biol Chem 277: 1981–1990. 10.1074/jbc.M108744200 11698407

[30] Sandmeier E, Hunziker P, Kunz B, Sack R, Christen P (1999) Spontaneous deamidation and isomerization of Asp108 in prion peptide 106-126 and in full-length prion protein. Biochem Biophys Res Commun 261: 578–583. 10441469 10.1006/bbrc.1999.1056

[31] Choi YC, Kim JL, Jeon YC, Park SJ, Choi EK, et al. (2004) Nonenzymatic glycation at the N terminus of pathogenic prion protein in transmissible spongiform encephalopathies. J Biol Chem 279 30402–30409. 10.1074/jbc.M400854200 15084583

[32] Gonzalez-Montalban N, Baskakov IV (2012) Assessment of strain-specific PrPSc elongation rates revealed a transformation of PrPSc properties during Protein Misfolding Cyclic Amplification. PLoS One 7: 0041210. 10.1371/journal.pone.0041210 PMC3398882 22815972

[33] Daus ML, Wagenfuhr K, Thomzig A, Boerner S, Hermann P, et al (2013) Infrared Microspectroscopy Detects Protein Misfolding Cyclic Amplification (PMCA)-Induced Conformational Alterations in Hamster Scrapie Progeny Seeds. J Biol Chem 288: 35068–35080. 24163371 10.1074/jbc.M113.497131 PMC3853259

[34] Gonzalez-Montalban N, Makarava N, Savtchenko R, Baskakov IV (2011) Relationship between Conformational Stability and Amplification Efficiency of Prions. Biochemistry 50: 7933–7940. 21848309 10.1021/bi200950v PMC3183828

[35] Makarava N, Kovacs GG, Savtchenko R, Alexeeva I, Budka H, et al (2011) Genesis of mammalian prions: from non-infectious amyloid fibrils to a transmissible prion disease. PLoS Pathogen 7: e1002419. 22144901 10.1371/journal.ppat.1002419 PMC3228811

[36] Makarava N, Kovacs GG, Savtchenko R, Alexeeva I, Ostapchenko VG, et al (2012) A New Mechanism for Transmissible Prion Diseases. J Neurosci 32: 7345–7355. 22623680 10.1523/JNEUROSCI.6351-11.2012 PMC3368278

[37] Castilla J, Gonzalez-Romero D, Saa P, Morales R, De Castro J, et al (2008) Crossing the Species Barrier by PrPSc Replication In Vitro Generates Unique Infectious Prions. Cell 134: 757–768. 18775309 10.1016/j.cell.2008.07.030 PMC2740631

[38] Green KM, Castilla J, Seward TS, Napier DL, Jewell JE, et al (2008) Accelerated High Fidelity Prion Amplification Within and Across Prion Species Barriers. PLOS Pathog 4: e1000139. 18769716 10.1371/journal.ppat.1000139 PMC2516356

[39] Gonzalez-Montalban N, Makarava N, Ostapchenko VG, Savtchenko R, Alexeeva I, et al (2011) Highly Efficient Protein Misfolding Cyclic Amplification. PLoS Pathogen 7: e1001277. 21347353 10.1371/journal.ppat.1001277 PMC3037363

[40] Aguzzi A, Nuvolone M, Zhu C (2013) The immunology of prion diseases. Nat Rev Immunology 13: 888–902. 24189576 10.1038/nri3553

[41] Mabbott NA (2012) Prion pathogenesis and secondary lymphoid organs. Prion 6: 322–333. 22895090 10.4161/pri.20676 PMC3609058

[42] Béringue V, Herzog L, Jaumain E, Reine F, Sibille P, et al (2012) Facilitated cross-species transmission of prions in extraneural tissue. Science 335: 472–475. 22282814 10.1126/science.1215659

[43] Heikenwalder M, Zeller N, Seeger H, Prinz M, Klohn PC, et al (2005) Chronic lymphocytic inflammation specifies the organ tropism of prions. Science 307: 1107–1110. 15661974 10.1126/science.1106460

[44] Heikenwalder M, Kurrer MO, Margalith I, Kranich J, Zeller N, et al (2008) Lymphotoxin-dependent prion replication in inflammatory stromal cells of granulomas. Immunity 29: 998–1008. 19100703 10.1016/j.immuni.2008.10.014

[45] Dron M, Moudjou M, Chapuis J, Salamat MKF, Bernand J, et al (2010) Endogenous Proteolytic Cleavage of Disease-associated Prion Protein to Produce C2 Fragments is strongly Cell- and Tissue-dependent. J Biol Chem 285: 10252–10264. 20154089 10.1074/jbc.M109.083857 PMC2856230

[46] Gonzalez-Montalban N, Lee YJ, Makarava N, Savtchenko R, Baskakov IV (2013) Changes in prion replication environemnt cause prion strain mutation. Faseb J 27: 3702–3710. 23729586 10.1096/fj.13-230466 PMC3752540

[47] Monti E, Bonten E, D'Azzo A, Bresciani R, Venerando B, et al (2010) Sialidases in vertebrates: A family of enzymes tailored for several cell functions. Adv Carbohydr Chem Biochem 64: 403–479. 20837202 10.1016/S0065-2318(10)64007-3

[48] Rosner H (1977) Gangliosides, sialoglycoproteins, and acetylcholinesterase of the developing mouse brain. Wilhelm Roux's Archives of Developmental Biology 183: 325–335. 10.1007/BF00848461 28304867

[49] Svennerholm L, Bostrom K, Jungbjer B (1997) Changes in weight and compositions of major mambrane components oh human brain during the span of adult human life of Swedes. Acta Neuropathol 94: 345–352. 9341935 10.1007/s004010050717

[50] Makarava N, Savtchenko R, Baskakov IV (2013) Selective amplification of classical and atypical prions using modified protein misfolding cyclic amplification J Biol Chem. 288: 33–41. 10.1074/jbc.M112.419531 PMC3537030 23168413

[51] Nishina K, Deleault NR, Mahal S, Baskakov I, Luhrs T, et al (2006) The Stoichiometry of Host PrPC Glycoforms Modulates the Efficiency of PrPSc formation in vitro. Biochemistry 45: 14129–14139. 17115708 10.1021/bi061526k

[52] Klingeborn M, Race B, Meade-White KD, Chesebro B (2011) Lower specific infectivity of protease-resistant prion protein generated in cell-free reactions. Proc Acad Natl Sci U S A 108: E1244–1253. 10.1073/pnas.1111255108 PMC3228482 22065744

[53] Tanaka K, Siwu ERO, Minami K, Hasegawa K, Nozaki S, et al (2010) Noninvasive Imaging of Dendrimer-Type N-Glycan Clusters: In Vivo Dynamics Dependence on Oligosaccharide Structure. Angew Chem Int Ed 49: 8195–8200. 10.1002/anie.201000892 PMC5484579 20857462

[54] Wang F, Wang X, Yuan CG, Ma J (2010) Generating a Prion Bacterially Expressed Recombinant Prion Protein. Science 327: 1132–1135. 20110469 10.1126/science.1183748 PMC2893558

[55] Deleault NR, Piro JR, Walsh DJ, Wang F, Ma J, et al (2012) Isolation of phosphatidylethanolamine as a solitary cofactor for prion formation in the absence of nucleic acids. Proc Acad Natl Sci U S A 109: 8546–8551. 10.1073/pnas.1204498109 PMC3365173 22586108

[56] Tuzi NL, Cancellotti E, Baybutt H, Blackford L, Bradford B, et al (2008) Host PrP Glycosylation: A Major Factor Determining the Outcome of Prion Infection. PLOS Biology 6: e100. 18416605 10.1371/journal.pbio.0060100 PMC2292751

[57] Varki A (1999) Sialic Acids. In: Varki A, Cummings R, Esko J, Freeze H, Hart G et al., editors. Essentials of Glycobiology. Cold Spring Harbor, NY: Cold Spring Harbor Laboratory Press. pp.195–210.

[58] Stamatos NM, Gomatos PJ, Cox J, Fowler A, Dow N, et al (1997) Desialylation of Peripheral Blood Mononuclear Cells Promotes Growth of HIV-1. Virology 228: 121–131. 10.1006/viro.1996.8373 9123818

[59] Stamatos NM, Carubelli I, van de Vlekkert D, Bonten EJ, Papini N, et al (2010) LPS-induced cytokine production in human dendritic cells is regulated by sialidase activity. J Leukoc Biol 88: 1227–1239. 20826611 10.1189/jlb.1209776 PMC2996894

[60] Miyagi T, Yamaguchi K (2012) Mammalian sialidases: physiological and pathological roles in cellular functions. Glycobiology 22: 880–896. 22377912 10.1093/glycob/cws057

[61] Bonten EJ, Campos Y, Zaitsev V, Nourse A, Waddell B, et al (2009) Heterodimerization of the sialidase NEU1 with the chaperone protective protein/cathepsin A prevents its premature oligomerization. J Biol Chem 284: 28430–28431. 19666471 10.1074/jbc.M109.031419 PMC2788892

[62] Lawson VA, Collins SJ, Masters CL, Hill AF (2005) Prion protein glycosylation. J Neurochem 93: 793–801. 15857383 10.1111/j.1471-4159.2005.03104.x

[63] Somerville RA (1999) Host and transmissible spongiform encephalopathy agent strain control glycosylation of PrP. J Gen Virol 80: 1865–1872. 10423157 10.1099/0022-1317-80-7-1865

[64] Parchi P, Giese A, Capellari S, Brown P, Schulz-Schaeffer W, et al (1999) Classification of sporadic Creutzfeldt-Jakob disease based on molecular and phenotypic analysis of 300 subjects. Ann Neurol 46: 224–233. 10443888

[65] Hill AF, Joiner S, Wadsworth JDF, Sidle KCL, Bell JE, et al (2003) Molecular classification of sporadic Creutzfeldt-Jakob disease. Brain 126: 1333–1346. 12764055 10.1093/brain/awg125

[66] Meyerett C, Michel B, Pulford B, Sparker TR, Nichols TA, et al (2008) In vitro strain adaptation of CWD prions by serial protein misfolding cyclic amplification. Virology 382: 267–276. 18952250 10.1016/j.virol.2008.09.023

[67] Barria MA, Telling GC, Gambetti P, Mastrianni JA, Soto C (2011) Generation of a new form of human PrPSc in vitro by interspecies transmission from cervid prions. J Biol Chem. 286: 7490–7495. 10.1074/jbc.M110.198465 PMC3045004 21209079

[68] Li J, Browning S, Mahal SP, Oelschlegel AM, Weissmann C (2010) Darwinian evolution of prions in cell culture. Science 327: 869–872. 20044542 10.1126/science.1183218 PMC2848070

[69] Browning S, Baker CA, Smith E, Mahal SP, Herva ME, et al (2011) Abrogation of Complex Glycosylation by Swainsonine Results in Strain- and Cell-specific Inhibition of Prion Replication J Biol Chem. 286: 40962–40973. 10.1074/jbc.M111.283978 PMC3220511 21930694

[70] Annunziata I, Patterson A, Helton D, Hu H, Moshiach S, et al (2013) Lysosomal NEU1 deficiency affects amyloid precursor protein levels and amyloid-b secretion via deregulated lysosomal exocytosis. Nat Commun 4: 2734. 24225533 10.1038/ncomms3734 PMC4015463

[71] Makarava N, Kovacs GG, Savtchenko R, Alexeeva I, Budka H, et al (2012) Stabilization of a prion strain of synthetic origin requires multiple serial passages. J Biol Chem 287: 30205–30214. 22807452 10.1074/jbc.M112.392985 PMC3436274

[72] de Geest N, Bonten E, Mann L, de Sousa-Hitzler J, Hahn C, et al (2002) Systemic and neurologic abnormalities distinguish the lysosomal disorders sialidosis and galactosialidosis in mice. Hum Mol Genet 11: 1455–1464. 12023988 10.1093/hmg/11.12.1455

