# Supplementary figures and images for: Sialylation of Prion Protein Controls the Rate of Prion Amplification, the Cross-Species Barrier, the Ratio of PrPSc Glycoform and Prion Infectivity
Source: PLoS Pathog. 2014 Sep 11;10(9):e1004366. doi: 10.1371/journal.ppat.1004366 (PMC4161476; doi:10.1371/journal.ppat.1004366)

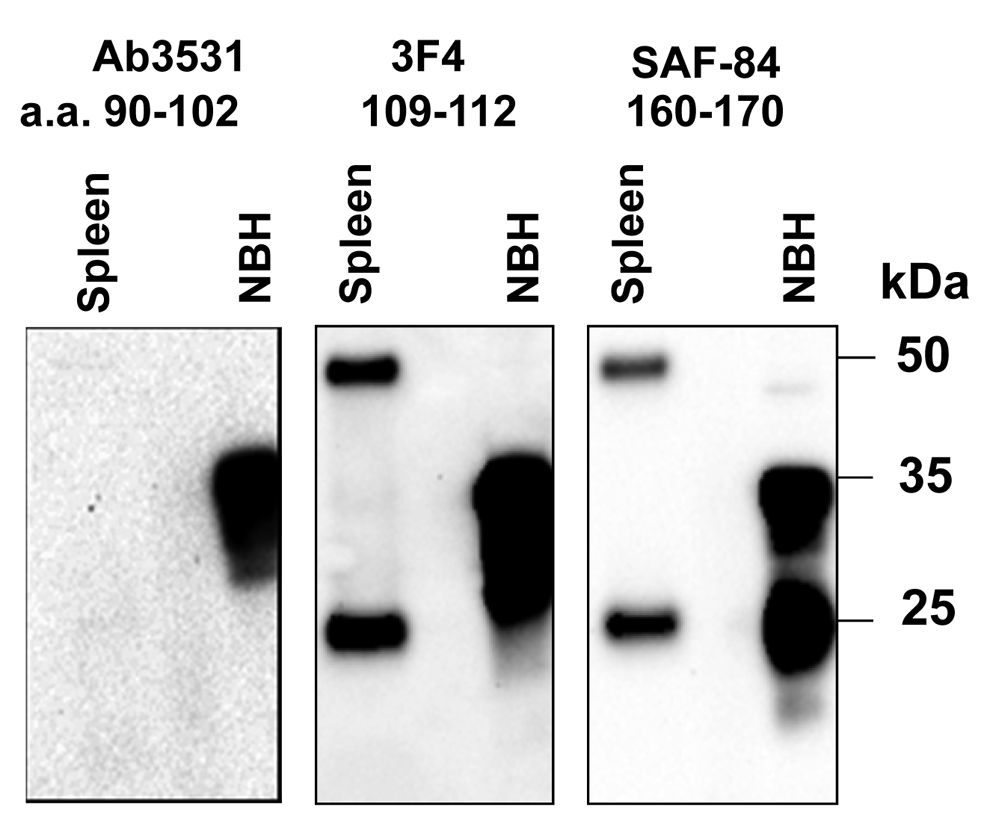

Supplement: Figure S1 — Western blot analysis of spleen- and brain-derived PrPC. Spleen- and brain-derived PrPC from non-infected animals was analyzed using Western blotting. The vast majority of spleen-derived PrPC appeared as a C2 proteolytic fragment at 25 kDa with its dimmer at 50 kDa. Blots were stained with Ab3531, 3F4 or SAF-84 antibodies as indicated. (TIF) [file ppat.1004366.s001.tif]

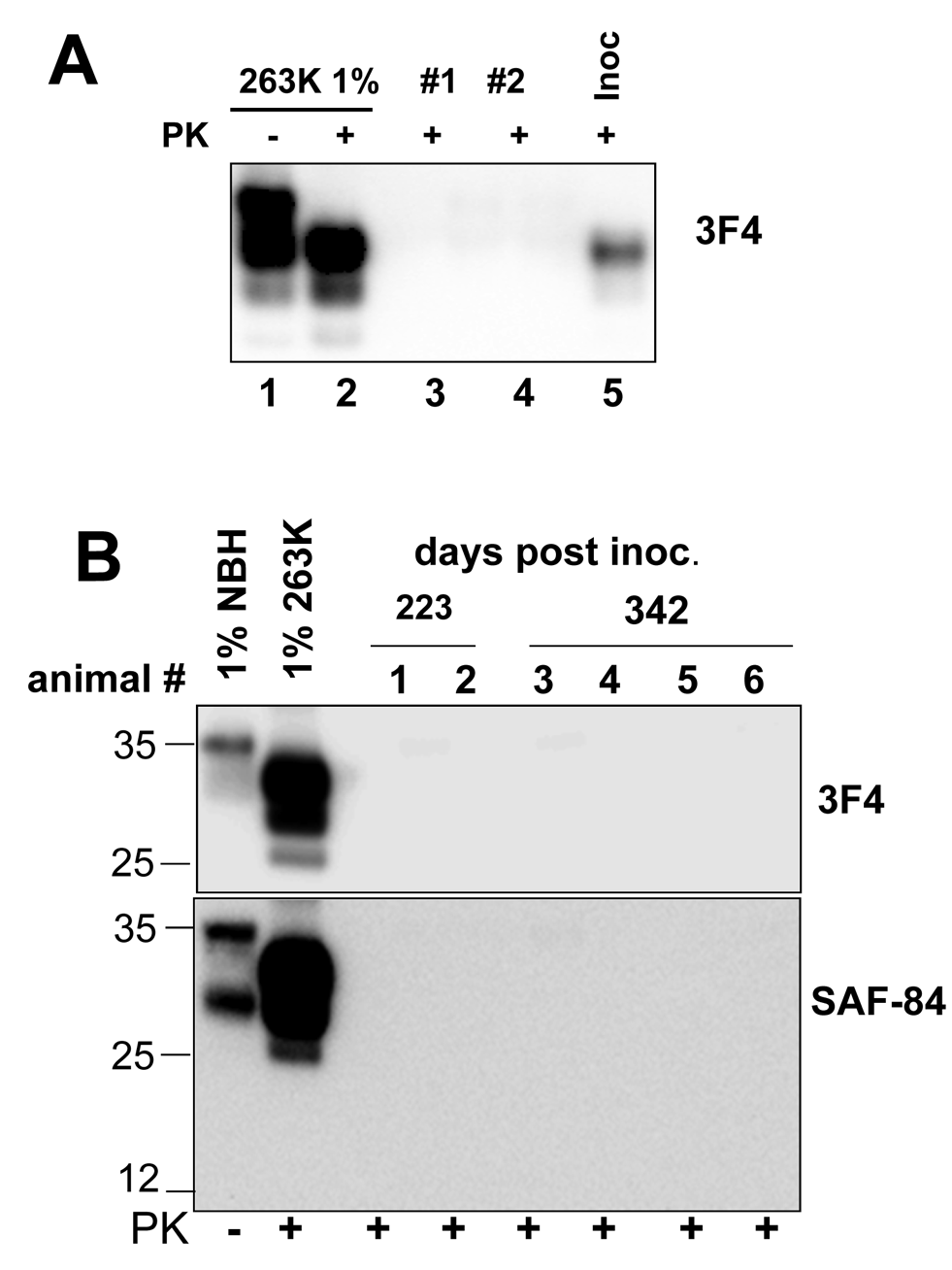

Supplement: Figure S2 — Western blot analysis of brain material from animals inoculated with de-sialylated PMCAb products. A. Animals #1 and 2 (lanes 3 and 4) were inoculated with PMCAb-derived materials produced using dsNBH. No clinical signs were developed and animal were euthanized at 223 days p.i. Initial inoculum is shown in lane 5; 1% 263K brain homogenate is shown as a reference in lane 2. B. Animals #1 to 6 were inoculated with PMCAb-derived materials produced using dsNBH and euthanized at 223 or 342 days p.i. Non-digested 1% NBH and PK-treated 1% 263K brain homogenates are shown as references. 3F4 or SAF-84 antibodies were used for staining. (TIF) [file ppat.1004366.s002.tif]

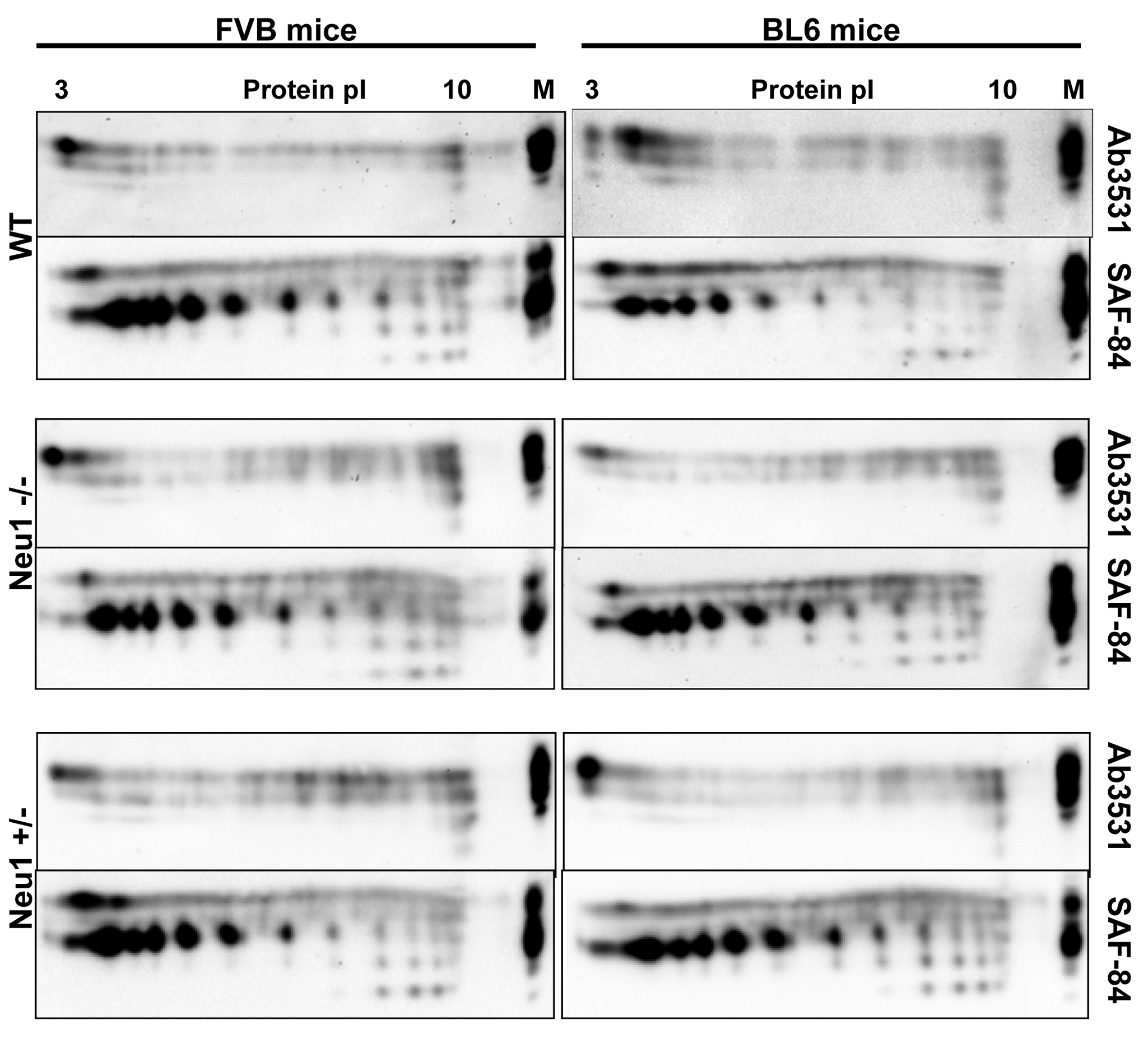

Supplement: Figure S3 — 2D analysis of PrPC and C1 in wild type, Neu1−/− and Neu1+/− mice. 2D analysis of 10% brain homogenates from wild type, Neu1−/− or Neu1+/− mice of FVB or BL6 genetic background. Blots were stained with Ab3531 or SAF-84 antibody, as indicated. M stands for a marker lane: brain samples were diluted 10-fold and used as references for 2D gels. (TIF) [file ppat.1004366.s003.tif]
